# Oropouche virus infection in patients with acute febrile syndrome: Is a predictive model based solely on signs and symptoms useful?

**DOI:** 10.1371/journal.pone.0270294

**Published:** 2022-07-26

**Authors:** Hilda V. Durango-Chavez, Carlos J. Toro-Huamanchumo, Wilmer Silva-Caso, Johanna Martins-Luna, Miguel Angel Aguilar-Luis, Juana del Valle-Mendoza, Zully M. Puyen

**Affiliations:** 1 School of Medicine, Universidad Peruana de Ciencias Aplicadas, Lima, Peru; 2 Universidad San Ignacio de Loyola, Unidad para la Generación y Síntesis de Evidencias en Salud, Lima, Peru; 3 Clínica Avendaño, Unidad de Investigación Multidisciplinaria, Lima, Peru; 4 School of Medicine, Research and Innovation Center of the Faculty of Health Sciences, Universidad Peruana de Ciencias Aplicadas, Lima, Peru; 5 Laboratorio de Biologia Molecular, Instituto de Investigación Nutricional, Lima, Peru; 6 Instituto Nacional de Salud, Lima, Peru; Stanford University School of Medicine, UNITED STATES

## Abstract

**Background:**

Oropouche fever is an infectious disease caused by the Oropouche virus (OROV). The diagnosis and prediction of the clinical picture continue to be a great challenge for clinicians who manage patients with acute febrile syndrome. Several symptoms have been associated with OROV virus infection in patients with febrile syndrome; however, to date, there is no clinical prediction rule, which is a fundamental tool to help the approach of this infectious disease.

**Objective:**

To assess the performance of a prediction model based solely on signs and symptoms to diagnose Oropouche virus infection in patients with acute febrile syndrome.

**Materials and methods:**

Validation study, which included 923 patients with acute febrile syndrome registered in the Epidemiological Surveillance database of three arbovirus endemic areas in Peru.

**Results:**

A total of 97 patients (19%) were positive for OROV infection in the development group and 23.6% in the validation group. The area under the curve was 0.65 and the sensitivity, specificity, PPV, NPV, LR + and LR- were 78.2%, 35.1%, 27.6%, 83.6%, 1.20 and 0.62, respectively.

**Conclusions:**

The development of a clinical prediction model for the diagnosis of Oropouche based solely on signs and symptoms does not work well. This may be due to the fact that the symptoms are nonspecific and related to other arbovirus infections, which confuse and make it difficult to predict the diagnosis, especially in endemic areas of co-infection of these diseases. For this reason, epidemiological surveillance of OROV in various settings using laboratory tests such as PCR is important.

## Introduction

Oropouche fever is an infectious disease caused by the Oropouche virus (OROV), which belongs to the *Orthobunyaviru*s genus [1,2] of the *Peribunyaviridae* family [3]. This virus is transmitted by arthropods, through the bite of the mosquito of the species *Culicoides paraensis* [4]. Direct human-to-human OROV transmission has not been demonstrated [5].

Since the first case of the disease caused by OROV was described in the year 1955 in Trinidad and Tobago [6], it is estimated that more than half a million individuals have been affected [7]. And since then, there have been 30 epidemics of Oropouche fever in the last 60 years in Latin American countries [7]. In the Americas, cases have been reported in countries such as Panama, Brazil [8], Peru [9],Ecuador, Trinidad y Tobago and French Guiana [10]. In Peru, the first outbreak of Oropouche fever was reported in Iquitos in 1992 [11].

The clinical symptoms of OROV fever are like those of other diseases caused by arboviruses such as Yellow Fever, Chikungunya (CHIKV), Zika (ZIKV), and Dengue (DENV) [12]. Acute febrile illness is a common manifestation in most patients with suspected arbovirus infection [13,14]. Also, coinfection is common when multiple viruses circulate [15]. Some research suggests that the symptoms of OROV fever are more like Dengue fever [15], with symptoms such as fever, headache, arthralgias, myalgias [16], dizziness, nausea, vomiting, which can be complicated by hemorrhagic manifestations [17], meningitis, and/or encephalitis [18]. Some patients have a morbilliform skin rash that resembles a dengue rash [12]. Furthermore, the incubation period for OROV fever is not precise, however, surveillance conducted during epidemics suggested that it might be between 4 to 8 days [4]. After this period, infected people present symptoms and develop high viremia, so they can transmit OROV if they are bitten by the vector mosquito [19]. The acute phase of the disease is between the first 3 to 5 days, an adequate time to collect blood samples from patients with suspected OROV infection and use them in diagnostic methods [4,12].

The laboratory diagnosis of OROV is carried out through molecular tests such as reverse transcription-polymerase chain reaction (RT-PCR) [12], real-time PCR (qRT-PCR) [19], isolation via cell culture [20], and serological tests with the detection of specific IgM antibodies from blood samples of people with suspected infection by OROV in the acute [4]. Although they have proven to be successful, they are still limited because they are not available in all countries [21], due to their high cost andthe lack of trained personnel to carry out the tests [14]. In Peru, arboviral diseases are etiologically identified by laboratory tests in less than 50% of cases, which leads to a scarce diagnosis of emerging arboviruses [15].

Therefore, it is necessary to develop a clinical prediction rule (CPR) that allows physicians to have an important weapon to direct the best treatment strategies [22] and to care for the population found in endemic areas due to arboviral diseases [23,24].

The objective of the present study was to develop and assess the performance of a predictive model based on signs and symptoms to diagnose Oropouche virus infection in patients with acute febrile syndrome.

## Materials and methods

### Study design and context

We conducted a secondary analysis of a database obtained in the framework of the project of epidemiological/syndromic surveillance of arbovirus diseases carried out by the Institute for Research and Nutrition, in accordance with the health directives of the National Center of Epidemiology, Prevention of Disease Control of the Ministry of Health of Peru from January 2015 to December 2016, in three areas from Peru [25].

Participants were from both sexes and with acute febrile syndrome from whom their blood sample were collected for the detection of arboviral diseases.

### Selection criteria

We included patients with acute febrile syndrome defined as axillary temperature ≥ 38°, with a duration ≤ 7 days, who attended a primary care center for medical care without an identifiable source of infection. Similarly, patients evaluated for the discard of the Oropouche virus (OROV) by molecular RT-PCR test were included, as well as patients diagnosed with other diseases such as DENV, ZIKV, CHIKV, Mayaro, *Leptospira* spp, *Rickettsia* spp.and *Bartonella* spp.using the molecular test RT-PCR. The patients who were not included in the present study were those with incomplete data or those did not have results for the laboratory test for OROV.

### Study population

We included data from patients with febrile syndrome with results of molecular RT-PCR tests for arboviral and bacterial diseases in three places (Puerto Maldonado, Piura, and Huánuco) in Peru during 2015–2016. All of them had to meet the selection criteria.

For the sample size, we based on the proposal of Riley RD et al (2020) [26]. Given that our outcome was the molecular diagnosis of OROV (binary outcome), we had to ensure a priori that the sample size was large enough to cover the following scenarios: a) Estimate the proportion of the outcome with adequate precision, b) aim for obtaining a small mean absolute prediction error, c) minimize the problem of "overfitting" and d) ensure a small difference in the model apparent and optimism adjusted R^2^ de Nagelkerke values.

An overall proportion of OROV equal to 26.4% was considered, and a total of nine predictors as possible candidates for the model, to cover the scenario of at least ten events for each predictor (in the development dataset there were a total of 97 events). Likewise, although the identification of OROV, in addition to the molecular test and serology, is basically focused on the identification of signs and symptoms [25], we decided to assume a conservative scenario, considering that our model would explain 30% of the variability. Thus, we define the maximum possible value of our $R_{cs}^{2}$ of our prediction model using the formula:

$$\max\left(R_{cs}^{2}\right)=1-exp\left(\frac{2\ln\;L_{null}}{n}\right)$$

In the same way, given that our outcome is binary, for our logistic regression model, the ln *L_null_* was defined under the formula:

$$\ln\;L_{null}=E\;\ln\left(\frac{E}{n}\right)+(n-E)\;\ln(1-\frac{E}{n}),$$

where *E* is the total number of individuals with the outcome present and *n* is an arbitrarily chosen sample size.

So, considering a proportion of the outcome of 0.30 (rounding to the decimal the 26.4% mentioned above), the maximum value of $R_{cs}^{2}$ was 0.71. Since we assumed that the new model would explain 30% or the variability, the anticipated value $R_{cs}^{2}$ was 0.30*0.71 = 0.213.

Considering the aforementioned, the extension ‘*pmsampsize’* was used in Stata v16.0 (StataCorp, TX, USA). Thus, further considering that the expected “shrinkage” required was only 10% (to minimize potential “overfitting”), it was obtained that it takes 334 individuals and a total of 89 events, to point to a mean absolute error in the predicted probabilities ≤0.05. This would cover scenarios 1, 3, and 4 mentioned above. To complement scenario 2, the same information was introduced to the online software https://mvansmeden.shinyapps.io/BeyondEPV/, resulting in a minimum required sample of 100 and a total of at least three (n = 3) events per variable.

### Variables

The outcome variable was the diagnosis of OROV, defined as a positive result of the molecular test for the Oropouche virus. The set of independent variables included sex, age in years, year of sample collection, and the different signs and symptoms.

### Data analysis

The Stata v16.0 software was used for the analysis. To do this, the overall sample was initially divided into two: a development group (DG) and a validation group (VG). The groups were randomized 2:1 using the Mersenne Twister method, taking as a primary reference the number of events (diagnosed cases of OROV).

A descriptive analysis stratified by DG and VG was presented. Since all the study variables were qualitative, they were presented as absolute and relative frequencies. In the bivariate analysis, to assess the association between the independent variables and OROV infection, only the DG was used. We follow statistical and epidemiological criteria for the potential initial prediction model. For the first, the variables that had a p <0.20 were considered. The variables of place and year of sampling, differential diagnoses, and signs/symptoms of ecchymosis, epistaxis, and hematuria were excluded a priori (the last one, because there were no positive cases at the crossings). Given that there were seven potential candidates, it was decided to add two under the epidemiological criteria: hyporexia (symptom) and petechiae (sign) [25]. For this crossing of variables, the Chi2 Test and Fisher’s Exact Test were used, according to the expected values.

For the multivariable analysis, using the development group, binary logistic regression was used with variances corrected by conglomerates (which were the three places the samples came from) to obtain odds ratios (ORs) with 95% confidence intervals (95% CI). First, the crude model was carried out for referential purposes. Subsequently, for the selection of the final variables, we performed a stepwise procedure (forward-backward) in successive rounds of analysis. In each round, the unselected variable that showed the lowest p-value in the previous round of the stepwise (forward step) was added to the model. Next, we remove the variables from the model if the previous step increased the p-value above the threshold (backward step). We stopped the procedure once all the unselected variables showed a p-value above the threshold in the working model. The presence of multicollinearity was assessed using procedures for the examination of the "conditioning" of the matrix of independent variables, as proposed by Belsey et al (1980), and using the coldiag2 command proposed by Hendrickx J (2004). All the "condition indexes" were less than 20, naturally,including the "condition number", which indicated the absence of collinearity problems.

The final multivariable model included the variables that provide a linear combination of these factors (weighted by regression parameters) associated with the outcome (OROV). Since the regression coefficients were mostly relatively similar, we decided to use equal factors (f = 1) in the final model to create a simple and easy-to-calculate score. A ROC (receiver operating characteristic) curve was constructed to determine a cut-off point above which the diagnosis of OROV could be predicted more reliably using the score generated. The choice of the best cut-off point was made with the Youden method and confidence intervals were generated using a bootstrapping technique of 100 repetitions. The goodness of fit of the model was evaluated with the Hosmer-Lemeshow test and a calibration graph of the observed versus predicted probabilities was constructed. The prediction model was externally validated in the validation dataset. The ability of the model to predict the diagnosis of OROV was evaluated through the area under the curve (AUC), using the estimates selected in the development base (training dataset). Finally, sensitivity, specificity, positive and negative predictive values, likelihood ratios, and diagnostic OR were calculated. For this, the cut-off point previously obtained was taken as a reference. These estimates were reported with a 95% confidence interval.

### Ethics

This research was approved by the Ethics Committee from *Universidad Peruana de Ciencias Aplicadas* in Lima, Peru (Document No: FCS-CEI/300-08-20). The samples were obtained in the context of the epidemiological/syndromic surveillance program in accordance with the health directives of the National Center for Epidemiology, Disease Control Prevention of the Ministry of Health of Peru after the participant gave their informed consent and authorization to obtain blood sample.

The information was only handled by the researchers and kept under the corresponding insurance, no person outside the investigation had access to the information.

## Results

**Fig 1:**
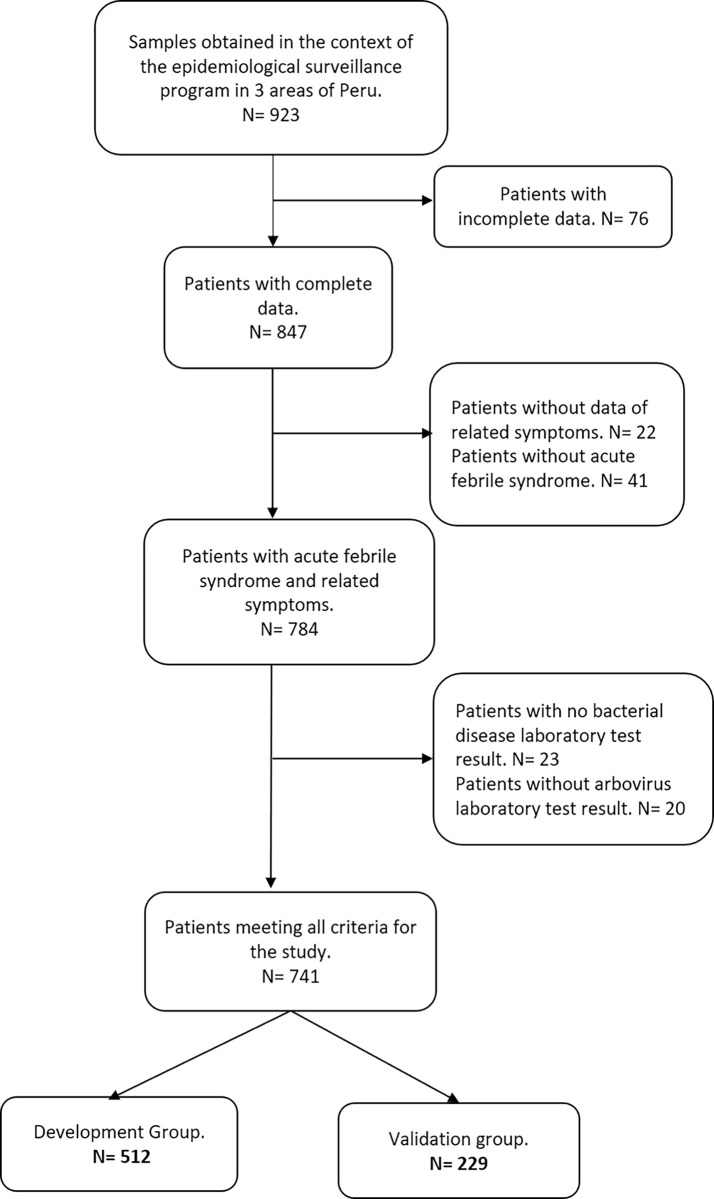
Enrollment flowchart.

In the present study, a total of 741 patients were included, who were randomly divided into 2 groups,using the method mentioned above, with the same composition of regions: Huánuco, Piura, and Puerto Maldonado. Thus, 512 were included in the development group (DG) and 229 in the validation group (VG) (Fig 1). In both groups, women were the majority of the population, 51.6% (n = 264) for DG and 50.7% (n = 116) for VG. For two groups, approximately 50% of the patients came from the Piura region. Likewise, 98% of the samples for the two groups were taken in 2016.

Regarding Oropouche infection, 19% (n = 97) of the patients were positive for DG and 23.6% (n = 54) for VG. Furthermore, positive patients were also detected in the two groups for DENV, CHIKV, ZIKV, Mayaro, *Bartonella* spp., *Rickettsia* spp. and *Leptospira* spp. Likewise, the most common symptoms that were evaluated in the patients who entered to the present study for the two groups (DG vs VG) were headache (92.6% vs 93.9%) followed by myalgia (83.6% vs 87.3%), arthralgia (80.5% vs 85.2%), retro-ocular pain (63.9% vs 64.6%), hyporexia (59.8% vs 63.3%) and odynophagia (32.0% vs 32.8%) (Table 1).

**Table 1:** Demographic and clinical characteristics of the participants in the development group and the validation group of a clinical prediction rule for Oropouche virus infection.

Characteristics		Development group† (n = 512)	Validation group† (n = 229)
**Sex**			
	Female	264 (51.5)	116 (50.7)
	Male	248 (48.5)	113 (49.3)
**Age (years)**			
	<15	150 (29.3)	51 (22.3)
	15–34	164 (32.0)	94 (41.0)
	35–54	119 (23.2)	49 (21.4)
	≥ 55	79 (15.4)	35 (15.3)
**Sample collection place**			
	Madre de Dios	63 (12.3)	39 (17.0)
	Huánuco	183 (35.7)	76 (33.2)
	Piura	266 (52.0)	114 (49.8)
**Year of sampling**			
	2015	8 (1.6)	4 (1.8)
	2016	504 (98.4)	225 (98.2)
**Oropouche**			
	Positive	97 (19.0)	54 (23.6)
	Negative	415 (81.0)	175 (76.4)
**Dengue**			
	Positive	134 (26.2)	57 (24.9)
	Negative	378 (73.8)	172 (75.1)
**Zika**			
	Positive	15 (2.9)	3 (1.3)
	Negative	497 (97.1)	226 (98.7)
**Chikungunya**			
	Positive	14 (2.7)	6 (2.6)
	Negative	498 (97.3)	223 (97.4)
**Mayaro**			
	Positive	44 (8.6)	22 (9.6)
	Negative	468 (91.4)	207 (90.4)
***Rickettsia* spp.**			
	Positive	37 (7.2)	19 (8.3)
	Negative	475 (92.8)	210 (91.7)
***Leptospira* spp.**			
	Positive	58 (11.3)	20 (8.7)
	Negative	454 (88.7)	209 (91.3)
***Bartonella* spp.**			
	Positive	11 (2.1)	9 (3.9)
	Negative	501 (97.9)	220 (96.1)
**Arthralgia**			
	Yes	412 (80.5)	195 (85.2)
	No	100 (19.5)	34 (14.8)
**Myalgia**			
	Yes	428 (83.6)	200 (87.3)
	No	84 (16.4)	29 (12.7)
**Headache**			
	Yes	474 (92.6)	215 (93.9)
	No	38 (7.4)	14 (6.1)
**Retro-ocular pain**			
	Yes	327 (63.9)	148 (64.6)
	No	185 (36.1)	81 (35.4)
**Hyporexia**			
	Yes	306 (59.8)	145 (63.3)
	No	206 (40.2)	84 (36.7)
**Odynophagia**			
	Yes	164 (32.0)	75 (32.8)
	No	348 (68.0)	154 (67.2)
**Skin rash**			
	Yes	104 (20.3)	44 (19.2)
	No	408 (79.7)	185 (80.8)
**Hematemesis**			
	Yes	5 (1.0)	3 (1.3)
	No	507 (99.0)	226 (98.7)
**Melena**			
	Yes	2 (0.4)	2 (0.9)
	No	510 (99.6)	227 (99.1)
**Epistaxis**			
	Yes	11 (2.2)	0 (0.0)
	No	501 (97.8)	229 (100.0)
**Gingivorrhagia**			
	Yes	4 (0.8)	4 (1.8)
	No	508 (99.2)	225 (98.2)
**Uterine Hemorrhage**			
	Yes	1 (0.2)	2 (0.9)
	No	511 (99.8)	227 (99.1)
**Petechiae**			
	Yes	11 (2.2)	6 (2.6)
	No	501 (97.8)	223 (97.4)
**Ecchymosis**			
	Yes	0 (0.0)	1 (0.4)
	No	510 (100.0)	228 (99.6)
**Haematuria**			
	Yes	0 (0.0)	0 (0.0)
	No	512 (100.0)	229 (100.0)
**Hemoptysis**			
	Yes	3 (0.6)	1 (0.4)
	No	509 (99.4)	228 (99.6)
**Severe abdominal pain**			
	Yes	18 (3.5)	19 (8.3)
	No	494 (96.5)	210 (91.7)
**Severe chest pain**			
	Yes	5 (1.0)	2 (0.9)
	No	507 (99.0)	227 (99.1)
**Persistent vomiting**			
	Yes	4 (0.8)	3 (1.3)
	No	508 (99.2)	226 (98.7)
**Hepatomegaly**			
	Yes	3 (0.6)	1 (0.4)
	No	509 (99.4)	228 (99.6)

† Randomized Groups 2:1 using Mersenne Twister.

In the bivariate analysis using the DG, of the demographic and clinical variables according to the molecular diagnosis of Oropouche in three regions of Peru, it was found a significant association for the diagnosis of Oropouche with the variables of age (p = 0.044), sample collection place (p <0.001), molecular diagnosis of *Leptospira* spp. (p = 0.004) and symptoms such as arthralgia (p <0.001), headache (p = 0.001), retro-ocular pain (p = 0.019) and odynophagia (p = 0.004) (Table 2).

**Table 2:** Demographic and clinical characteristics of the participants who belong to the development group of a clinical prediction rule according to the presence of Oropouche virus infection.

Characteristics		Oropouche		p
		Yes (n = 97)	No (n = 415)	
**Sex**				0.113
	Female	43 (16.3)	221 (83.7)	
	Male	54 (21.8)	194 (78.2)	
**Age (years)**				0.044
	<15	39 (26.0)	11 (74.0)	
	15–34	25 (15.2)	139 (84.8)	
	35–54	17 (14.3)	102 (85.7)	
	≥ 55	16 (20.3)	63 (79.7)	
**Sample collection place**				<0.001
	Madre de Dios	2 (3.2)	61 (96.8)	
	Huánuco	30 (16.4)	153 (83.6)	
	Piura	65 (24.4)	201 (75.6)	
**Year of sampling**				0.650† †
	2015	2 (25.0)	6 (75.0)	
	2016	95 (18.9)	409 (91.1)	
**Dengue**				0.722
	Positive	24 (17.9)	110 (82.1)	
	Negative	73 (19.3)	305 (80.7)	
**Zika**				0.176† †
	Positive	5 (33.3)	10 (66.7)	
	Negative	92 (18.5)	405 (81.5)	
**Chikungunya**				0.083† †
	Positive	0 (0.0)	14 (100.0)	
	Negative	97 (19.5)	401 (80.5)	
**Mayaro**				0.061
	Positive	13 (29.6)	31 (70.4)	
	Negative	84 (17.9)	384 (82.1)	
***Rickettsia* spp.**				0.19
	Positive	4 (10.8)	33 (89.2)	
	Negative	93 (19.6)	382 (80.4)	
***Leptospira* spp.**				0.004
	Positive	19 (32.8)	39 (67.2)	
	Negative	78 (17.2)	376 (82.8)	
***Bartonella* spp.**				0.136† †
	Positive	0 (0.0)	11 (100.0)	
	Negative	97 (19.4)	404 (80.6)	
**Arthralgia**				< 0.001
	Yes	63 (15.3)	349 (84.7)	
	No	34 (34.0)	66 (66.0)	
**Myalgia**				0.064
	Yes	75 (17.5)	353 (82.5)	
	No	22 (26.2)	62 (73.8)	
**Headache**				0.001
	Yes	82 (17.3)	392 (82.7)	
	No	15 (39.5)	23 (60.5)	
**Retro-ocular pain**				0.019
	Yes	52 (15.9)	275 (84.1)	
	No	45 (24.3)	140 (75.7)	
**Hyporexia**				0.354
	Yes	62 (20.3)	244 (79.7)	
	No	35 (17.0)	171 (83.0)	
**Odynophagia**				0.004
	Yes	43 (26.2)	121 (73.8)	
	No	54 (15.5)	294 (84.5)	
**Skin rash**				0.228
	Yes	24 (23.1)	80 (76.9)	
	No	73 (17.9)	335 (82.1)	
**Hematemesis**				0.589† †
	Yes	0 (0.0)	5 (100.0)	
	No	97 (19.1)	410 (80.9)	
**Melena**				1.000† †
	Yes	0 (0.0)	2 (100.0)	
	No	97 (19.0)	413 (81.0)	
**Epistaxis**				0.136† †
	Yes	0 (0.0)	11 (100.0)	
	No	97 (19.4)	404 (80.6)	
**Gingivorrhagia**				1.000† †
	Yes	0 (0.0)	4 (100.0)	
	No	97 (19.1)	411 (80.9)	
**Uterine Hemorrhage**				1.000† †
	Yes	0 (0.0)	1 (100.0)	
	No	97 (19.0)	414 (81.0)	
**Petechiae**				0.699† †
	Yes	1 (9.1)	10 (90.9)	
	No	96 (19.2)	405 (80.8)	
**Ecchymosis**				NA
	Yes	0 (0.0)	0 (0.0)	
	No	97 (19.0)	415 (81.0)	
**Haematuria**				NA
	Yes	0 (0.0)	0 (0.0)	
	No	97 (19.0)	415 (81.0)	
**Hemoptysis**				0.468† †
	Yes	1 (33.3)	2 (66.7)	
	No	96 (18.9)	413(81.1)	
**Severe abdominal pain**				0.547† †
	Yes	2 (11.1)	16 (88.9)	
	No	95 (19.2)	399 (80.8)	
**Severe chest pain**				0.241† †
	Yes	2 (40.0)	3 (60.0)	
	No	95 (18.7)	412 (81.3)	
**Persistent vomiting**				0.570† †
	Yes	1 (25.0)	3 (75.0)	
	No	96 (18.9)	412 (81.1)	
**Hepatomegaly**				1.000† †
	Yes	0 (0.0)	3 (100.0)	
	No	97 (19.1)	412 (80.9)	

NA: Not applicable due to no observations in any category.

* Molecular diagnosis by PCR.

† p value of Chi-square test.

† † p value of Fisher’s exact test.

Of the nine variables initially considered as candidates for the model, arthralgia, headache, odynophagia, and petechiae were finally selected after the stepwise procedure (Table 3). However, odynophagia was the only variable with an OR > 1. This gave an initial view that the consideration of signs and symptoms would be insufficient to generate a prediction model. For practical purposes, we opted to consider the four variables previously mentioned.

**Table 3:** Crude and adjusted logistic regression models for the presence of Oropouche virus infection in the development group of a clinical prediction rule.

Variables	Crude model†			Adjusted††		
	OR*	CI 95%	p	OR	CI 95%	p
**Male sex**	1.43	0.72–2.83	0.304			
**Age (Ref: < 15 years)**						
15–34 years	0.51	0.40–0.66	<0.001			
35–55 years	0.47	0.32–0.71	<0.001			
≥ 55 years	0.72	0.68–0.77	<0.001			
**Arthralgia**	0.35	0.28–0.43	<0.001	0.39	0.32–0.49	**<0.001**
**Myalgia**	0.60	0.50–0.72	<0.001			
**Headache**	0.32	0.25–0.42	<0.001	0.41	0.35–0.47	**<0.001**
**Retro-ocular pain**	0.59	0.33–1.05	0.072			
**Odynophagia**	1.93	1.30–2.89	0.001	2.07	1.40–3.06	**<0.001**
**Hyporexia**	1.24	0.93–1.65	0.138			
**Petechiae**	0.42	0.19–0.92	0.03	0.40	0.18–0.87	**0.022**

* OR: Odds ratio CI 95%: Confidence interval at 95%.

† Logistic Regression with variances corrected for clusters (three sites where the samples come from) in crude model

†† Logistic Regression with variances corrected for clusters (three places where the samples come from) adjusted for the variables that were entered according to the stepwise method (forward-backward). Criteria for backward and forward from the model were p <0.05 and p≥0.20, respectively.

**Fig 2:**
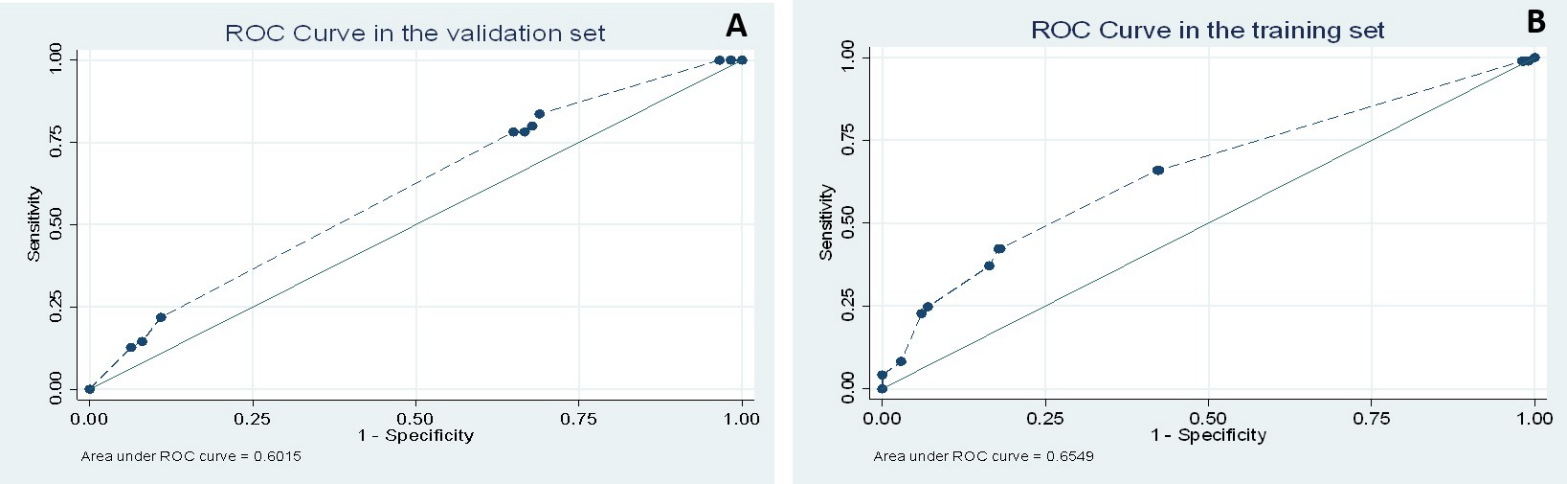
(A) ROC curve (Receiver Operating Characteristics) of the Development Group of the clinical prediction rule for infection by Oropouche Virus. (B) ROC curve of the Validation Group of the clinical prediction rule for infection by Oropouche Virus.

**Fig 3:**
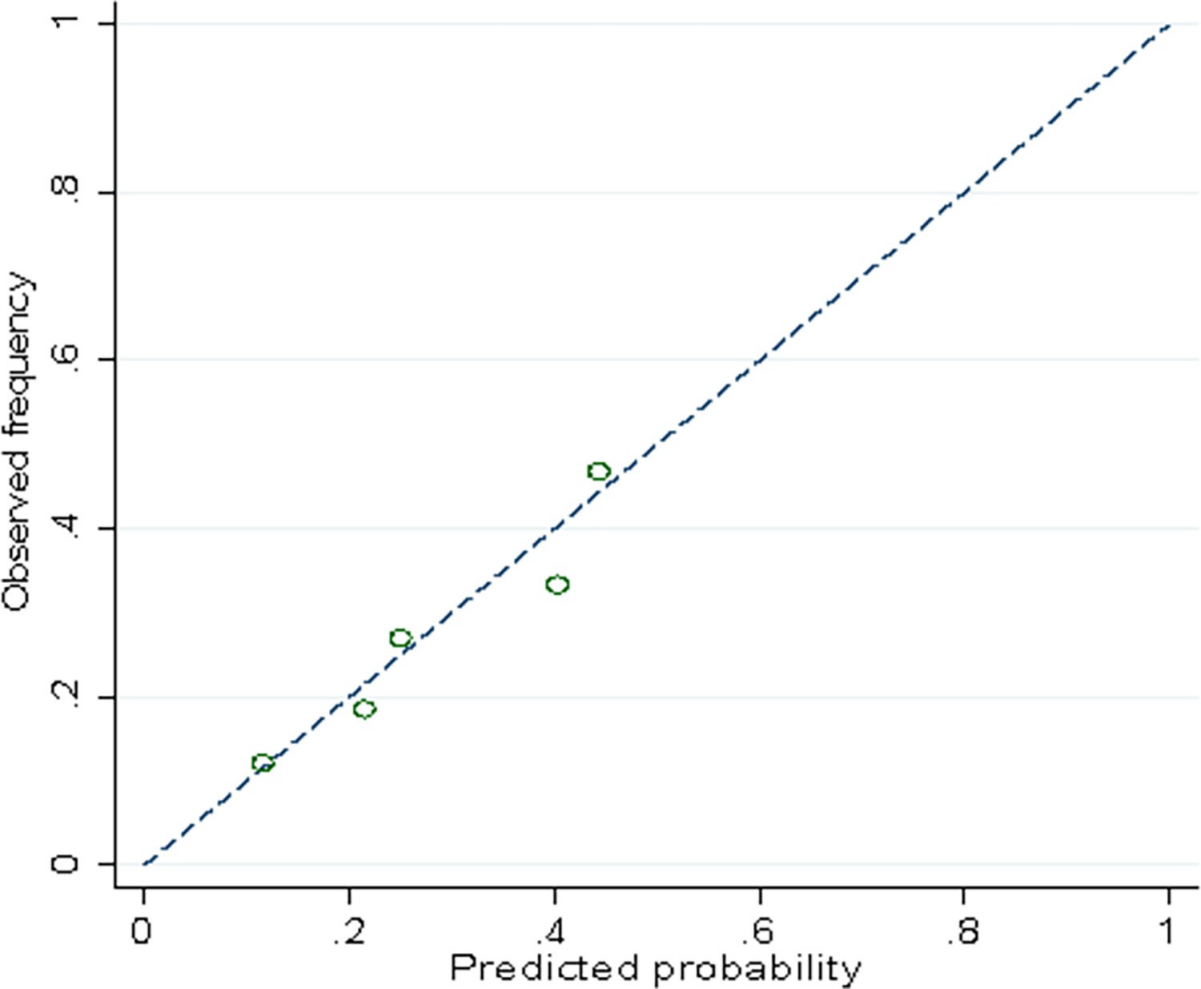
Calibration graph of the clinical prediction model for infection by Oropouche.

To simplify the calculations, we used equal factors (f = 1) for the overall score, ignoring the variations between the regression coefficients. The model obtained an area under the curve (AUC) of 0.65 (95% CI: 0.59 to 0.71) (Fig 2A). The Hosmer-Lemeshow test showed good agreement between the observed and predicted probabilities, with a goodness of fit p-value of 0.78 (Fig 3).

Discrimination remained low in the VG with an AUC of 0.60 (95% CI: 0.53 to 0.68) (Fig 2B). The maximized value of the Youden index performed with the Bootstrap method was -1.2 (95% CI:-1.66 to -0.77). The sensitivity was 78.2% (95% CI: 65.0 to 88.2) and the specificity was 35.1% (95% CI: 28.0 to 42.6) (Table 4). The low specificity and low likeliyhood ratios confirmed that a prediction model would not be useful.

**Table 4:** Sensitivity, specificity, predictive values, and likelihood indices for the cut-off point defined for the clinical prediction rule for Oropouche in the validation group.

Tests	Value	CI 95%†	
		Lower limit	Upper limit
**Prevalence**	24%	18.60%	30.10%
**Sensitivity**	78%	65.00%	88.20%
**Specificity**	35.10%	28%	42.60%
**Positive Predictive Value**	27.60%	20.70%	35.30%
**Negative Predictive Value**	84%	73.00%	91.20%
**Positive Likelihood Ratio**	1.2	1.01	1.44
**Negative Likelihood Ratio**	0.62	0.36	1.07
**Odds Ratio**	1.93	0.96	3.9

† CI 95%: Confidence interval at 95%.

## Discussion

For the present study, the area under the curve (AUC) of CPR was close to 0.5, which means that it has low discriminatory power and low predictive capacity to classify sick from healthy patients. In other words, the AUC of our CPR is closer to non-discrimination than to perfection. Therefore, the clinical prediction rule might not necessarily be as acceptable to discriminate patients with and without Oropouche.

The prediction model based only on signs and symptoms did not prove to be useful or to have a good discriminatory capacity. This is because the symptoms of the patient with Oropouche fever were very similar to those of other arboviral diseases and not specific to Oropouche infection [5]. This does not help with the prediction or diagnosis of OROV disease [3], especially in endemic areas of coinfection of these diseases [5,27]. The findings of this study are similar to other studies that described the typical clinical manifestations such as fever, headache, myalgia, arthralgia. [4], retroocular pain, odynophagia, and hyporexia [25] but were unable to develop clinical prediction rules.

In the population studied, the most frequent signs and symptoms were headaches, followed by myalgia, arthralgia, retro-ocular pain, hyporexia, and odynophagia. Similar results were identified by Mourão et al (2015) who described that the most frequent symptoms were headache (72.7%), myalgia (70.3%), and arthralgia (57.8%) in 631 patients who were tested by serological tests [16]. A review of Travassos Da Rosa JF et al (2017) reported that the most frequent symptoms that accompany the acute febrile illness caused by OROV were headache, myalgia, arthralgia, anorexia, chills, dizziness, and photophobia [1].

In our development and validation groups, 19% and 23.6% had a positive diagnosis for OROV, respectivelyt. Likewise, the presence of co-infections was detected, the most frequent being associated with the DENV. Different results were reported in the research conducted by Wise et al. (2020), in Ecuadorian patients with undifferentiated febrile illness, where using qRT-PCR they detected positive cases of OROV (3.1%, n = 6), DENV (2.0%, n = 4), ZIKV(11.2%, n = 22) and *Leptospira* spp. (0.51%, n = 1) [19].

In the same way, the variable odynophagia was the only one that was positively associated with the clinical diagnosis of OROV using the CPR. Therefore, symptomatology is probably not useful to build a clinical prediction model, especially in a country like Peru, in which there is a high prevalence of arboviral diseases that end up overlapping because they are differential diagnoses [27,28]. These findings are consistent with other studies where it was found that the etiological clinical diagnosis of OROV infection was not possible due to the low specificity of the symptoms, which is why molecular diagnosis is recommended [15,27,28].

In the absence of OROV prediction based solely on symptoms, laboratory tests like RT-PCR can provide specific and accurate diagnosis. In Peru and other similar locations where other arboviral diseases are endemic, the OROV may be underestimated [25]. Therefore, the diagnosis of OROV must be based on specific laboratory tests that confirm the infection by OROV [3]

Studies show that (RT-PCR) is the most sensitive and specific test for the detection of RNA via nuclear amplification directly from clinical samples [18,19]. Although this method depends on the day of clinical disease [28].The rate of detection of OROV RNA during the first five days of illness was 93.3% using qRT-PCR [20]. The acute phase of the sickness lasts 3 to 5 days, which is enough time to collect blood samples from OROV-infected patients and use them in diagnostic procedures [4,12]. So, the number of days since you first became ill may aid in disease diagnosis or increase the accuracy of laboratory test results.

At the epidemiological level, the Peruvian National System has not proposed any detection strategy for the laboratory diagnosis of the OROV virus [25]. In primary care centers, it is a challenge for physicians to differentiate OROV fever from other diseases that are circulating in endemic areas, especially DENV, ZIKV and CHIKV.Therefore it is recommended that the differential clinical diagnosis be based on specific laboratory tests that confirm OROV infection [27]. This will improve decision making in cases of clinical uncertainty and improve the diagnostic, therapeutic, or prognostic precision of arbovirus infections [29]. Likewise, limited importance has been given to the surveillance of this disease due to the lack of notification of health centers and hospitals, which leads to to its lack of diagnosis as the main cause, which leads to the number of reported cases being low, especially in endemic regions [30]. Previous research indicated that it is relevant to include the clinical and laboratory diagnosis of OROV in epidemiological surveillance [30]. Especially in Peru, to propose the best mitigation strategies for these diseases, and to appropriately allocate public health resources for the use of sophisticated and highly efficient laboratory tests [17,27]. Therefore, to have a highly efficient detection of OROV, it is essential to invest in the equipment required for the molecular diagnosis of OROV and to train the professionals who will carry out the tests [28].

Likewise, the associations found in our study highlight the importance of implementing the most sensitive and specific clinical and laboratory diagnoses for the detection of OROV in the epidemiological surveillance of Peru.

Finally, it is important to mention that the samples of our study were collected during the cyclical meteorological phenomenon of "El Niño", which could imply an increase in the incidence and resurgence of some infections responsible for the acute febrile syndrome in Peru [31]. Likewise, globally, it has been described that climate and environmental change, meteorological phenomena, and the migration of people and/or animals are the main factors for the appearance of arboviral diseases and a driver of the appearance of OROV, which represent a threat to public health [5]. In addition, studies suggest that anthropogenic changes in the soil (road construction, deforestation) have a negative impact on the habitat of the hosts, forcing them to move closer to urban regions, favoring the spread of vectors [4,5,11]. In the present study, most of the samples taken were in Piura with 50%, considered an endemic area for arboviral diseases. Martins-Luna J et al. identified that outbreaks of different arboviruses and zoonotic diseases coexisted in this region of Peru in the years 2015–2016 [25].

This study has some limitations. First, we used a secondary database elaborated in the context of epidemiological surveillance, so the data was taken in the context of endemic areas and at outbreak periods, which limits the application of our prediction rule to these contexts. Second, there was no data on the time in days of the febrile syndrome where the blood samples were taken for evaluation, which could have been an important variable in the elaboration of the rule. Sakkas et al., found that the incubation period is between 3 to 8 days, and the diagnosis of OROV has a higher sensitivity in the first 5 days, which may influence the initial diagnosis of OROV [5].

## Conclusions

In conclusion, the development of a clinical prediction model for the diagnosis of Oropouche based solely on signs and symptoms does not work well. Predictive symptomatological variables are not useful to build a model of clinical prediction, especially in areas with a high prevalence of other arboviral diseases that make the clinical diagnosis of OROV difficult. This may be due to the fact that the symptoms are nonspecific and related to other arbovirals, which confuses and makes it difficult to predict the diagnosis of patients with OROV, especially in endemic areas of coinfection of arboviral diseases [25]. The early confirmation of the diagnosis of OROV is also essential for health professionals to have tools for decision-making in the management of a patient with symptoms related to arboviral diseases and for the efficient management of public health resources destined for the control of vectors [28]. Surveillance for Oropouche fever should not be limited to endemic regions and outbreak times [25]. Therefore, cases of OROV should be reported under routine conditions and in non-endemic areas, to better understand the dynamics of arboviral diseases, especially Oropouche fever. It is essential that the epidemiological surveillance of OROV is carried out using laboratory tests such as RT-PCR or serology.

## Supporting information

S1 Table Dataset of Oropouche virus infection in patients with acute febrile syndrome. (XLSX)

## References

[1] Travassos Da Rosa JF, De Souza WM, De Paula Pinheiro F, Figueiredo ML, Cardoso JF, Acrani GO, et al. Oropouche virus: Clinical, epidemiological, and molecular aspects of a neglected orthobunyavirus. Am J Trop Med Hyg. 2017;96(5):1019–30. doi: 10.4269/ajtmh.16-0672 28167595 PMC5417190

[2] Ladner JT, Savji N, Lofts L, da Rosa AT, Wiley MR, Gestole MC, et al. Genomic and phylogenetic characterization of viruses included in the Manzanilla and Oropouche species complexes of the genus Orthobunyavirus, family Bunyaviridae. J Gen Virol. 2014;95(PART 5):1055–66. doi: 10.1099/vir.0.061309-0 24558222 PMC5974287

[3] Silva-Caso W, Aguilar-Luis MA, Palomares-Reyes C, Mazulis F, Weilg C, del Valle LJ, et al. First outbreak of Oropouche Fever reported in a non-endemic western region of the Peruvian Amazon: Molecular diagnosis and clinical characteristics. Int J Infect Dis. 2019 Jun 1;83:139–44. doi: 10.1016/j.ijid.2019.04.011 30991139

[4] Romero-Alvarez D, Escobar LE. Oropouche fever, an emergent disease from the Americas. Microbes Infect [Internet]. 2018 Mar 1 [cited 2020 Jun 22];20(3):135–46. Available from: https://pubmed.ncbi.nlm.nih.gov/29247710/. doi: 10.1016/j.micinf.2017.11.013 29247710

[5] Sakkas H, Bozidis P, Franks A, Papadopoulou C. Oropouche fever: A review. Viruses [Internet]. 2018 Apr 1 [cited 2020 Jun 14];10(4):175. Available from: https://www.ncbi.nlm.nih.gov/pmc/articles/PMC5923469/. doi: 10.3390/v10040175 29617280 PMC5923469

[6] Anderson CR, Spence L, Downs WG, Aitken TH. Oropouche virus: a new human disease agent from Trinidad, West Indies. Am J Trop Med Hyg. 1961 Jul;10:574–8. doi: 10.4269/ajtmh.1961.10.574 13683183

[7] Vasconcelos HB, Nunes MRT, Casseb LMN, Carvalho VL, da Silva EVP, Silva M, et al. Molecular epidemiology of oropouche virus, Brazil [Internet]. Vol. 17, Emerging Infectious Diseases. Centers for Disease Control and Prevention; 2011 [cited 2020 Oct 28]. p. 800–6. Available from: /pmc/articles/PMC3321770/?report = abstract. 10.3201/eid1705.101333 PMC3321770 21529387

[8] Tilston-Lunel NL, Hughes J, Acrani GO, da Silva DEA, Azevedo RSS, Rodrigues SG, et al. Genetic analysis of members of the species Oropouche virus and identification of a novel M segment sequence. J Gen Virol [Internet]. 2015 Jul 1 [cited 2020 Jun 22];96(7):1636–50. Available from: https://www.ncbi.nlm.nih.gov/pmc/articles/PMC4635451/. doi: 10.1099/vir.0.000108 25735305 PMC4635451

[9] Gutierrez B, Wise EL, Pullan ST, Logue CH, Bowden TA, Escalera-Zamudio M, et al. Evolutionary Dynamics of Oropouche Virus in South America. J Virol [Internet]. 2019 Dec 4 [cited 2020 Jun 23];94(5). Available from: https://www.ncbi.nlm.nih.gov/pmc/articles/PMC7022353/. 10.1128/JVI.01127-19 PMC7022353 31801869

[10] World Health Organization. Oropouche virus disease—French Guiana [Internet]. 2020 [cited 2021 Jul 4]. Available from: https://www.who.int/emergencies/disease-outbreak-news/item/oropouche-virus-disease—french-guiana-france.

[11] Romero-Alvarez D, Escobar LE. Vegetation loss and the 2016 Oropouche fever outbreak in Peru. Mem Inst Oswaldo Cruz. 2017 Apr 1;112(4):292–8. doi: 10.1590/0074-02760160415 28327792 PMC5354615

[12] Endy TP. Viral Febrile Illnesses and Emerging Pathogens. In: Hunter’s Tropical Medicine and Emerging Infectious Diseases [Internet]. Elsevier; 2020 [cited 2020 Jun 22]. p. 325–50. Available from: https://www.ncbi.nlm.nih.gov/pmc/articles/PMC7151808/.

[13] Moreira J, Bressan CS, Brasil P, Siqueira AM. Epidemiology of acute febrile illness in Latin America. Clin Microbiol Infect [Internet]. 2018 Aug 1 [cited 2020 Jun 23];24(8):827–35. Available from: https://www.ncbi.nlm.nih.gov/pmc/articles/PMC7172187/. doi: 10.1016/j.cmi.2018.05.001 29777926 PMC7172187

[14] Sippyid R, Farrell DF, Lichtenstein DA, Nightingale R, Harris MA, Toth J, et al. Severity index for suspected arbovirus (SISA): Machine learning for accurate prediction of hospitalization in subjects suspected of arboviral infection. PLoS Negl Trop Dis [Internet]. 2020 Feb 1 [cited 2020 Jun 23];14(2). Available from: https://www.ncbi.nlm.nih.gov/pmc/articles/PMC7046343/. 10.1371/journal.pntd.0007969 PMC7046343 32059026

[15] Alva-Urcia C, Aguilar-Luis MA, Palomares-Reyes C, Silva-Caso W, Suarez-Ognio L, Weilg P, et al. Emerging and reemerging arboviruses: A new threat in Eastern Peru. PLoS One. 2017 Nov 1;12(11). doi: 10.1371/journal.pone.0187897 29136650 PMC5685628

[16] Mourão MPG, De Bastos MS, de Figueiredo RMP, De Gimaque JBL, Alves V do CR, Saraiva M das GG, et al. Arboviral diseases in the western brazilian amazon: A perspective and analysis from a tertiary health & research center in manaus, state of Amazonas. Rev Soc Bras Med Trop [Internet]. 2015 [cited 2020 Jun 22];48:20–6. Available from: https://pubmed.ncbi.nlm.nih.gov/26061367/. doi: 10.1590/0037-8682-0133-2013 26061367

[17] Alvarez-Falconi PP, Amanda B, Ruiz R. Brote de Fiebre de Oropuche en Bagazán, San Martín—Perú: Evaluación Epidemiológica, Manifestaciones Gastrointestinales y Hemorrágicas [Internet]. Vol. 30, Rev. Gastroenterol. Perú. 2010 Sep [cited 2021 Jul 4]. Available from: http://www.revistagastroperu.com/index.php/rgp/article/view/421. 21263761

[18] Vernal S, Martini CCR, da Fonseca BAL. Oropouche virus–associated aseptic meningoencephalitis, Southeastern Brazil. Emerg Infect Dis [Internet]. 2019 Feb 1 [cited 2020 Jun 22];25(2):380–2. Available from: https://www.ncbi.nlm.nih.gov/pmc/articles/PMC6346467/. doi: 10.3201/eid2502.181189 30666950 PMC6346467

[19] Wise EL, Márquez S, Mellors J, Paz V, Atkinson B, Gutierrez B, et al. Oropouche virus cases identified in Ecuador using an optimised qrt-pcr informed by metagenomic sequencing. PLoS Negl Trop Dis [Internet]. 2020 Jan 1 [cited 2020 Oct 28];14(1):1–15. Available from: https://www.ncbi.nlm.nih.gov/pmc/articles/PMC6994106/. doi: 10.1371/journal.pntd.0007897 31961856 PMC6994106

[20] Weidmann M, Rudaz V, Nunes MRT, Vasconcelos PFC, Hufert FT. Rapid detection of human pathogenic orthobunyaviruses. J Clin Microbiol [Internet]. 2003 Jul 1 [cited 2021 Jul 4];41(7):3299–305. Available from: /pmc/articles/PMC165340/. doi: 10.1128/JCM.41.7.3299-3305.2003 12843078 PMC165340

[21] Crump JA, Gove S, Parry CM. Management of adolescents and adults with febrile illness in resource limited areas. BMJ [Internet]. 2011 Aug 13 [cited 2021 Jul 6];343(7819). Available from: /pmc/articles/PMC3164889/. doi: 10.1136/bmj.d4847 21824901 PMC3164889

[22] Cowley LE, Farewell DM, Maguire S, Kemp AM. Methodological standards for the development and evaluation of clinical prediction rules: a review of the literature. Diagnostic Progn Res [Internet]. 2019 Dec [cited 2020 Jul 15];3(1). Available from: https://www.ncbi.nlm.nih.gov/pmc/articles/PMC6704664/. doi: 10.1186/s41512-019-0060-y 31463368 PMC6704664

[23] Ban JW, Emparanza JI, Urreta I, Burls A. Design characteristics influence performance of clinical prediction rules in validation: A meta-epidemiological study. PLoS One [Internet]. 2016 Jan 5 [cited 2020 Jul 15];11(1). Available from: https://www.ncbi.nlm.nih.gov/pmc/articles/PMC4701404/. doi: 10.1371/journal.pone.0145779 26730980 PMC4701404

[24] Laupacis A, Sekar N, Stiell LG. Clinical Prediction Rule. A Review and Suggested Modifications of Methodological Standards. JAMA J Am Med Assoc [Internet]. 1997 Feb 12 [cited 2020 Jul 13];277(6):488–94. Available from: https://jamanetwork.com/journals/jama/fullarticle/414118. 9020274

[25] Martins-Luna J, Del Valle-Mendoza J, Silva-Caso W, Sandoval I, Del Valle LJ, Palomares-Reyes C, et al. Oropouche infection a neglected arbovirus in patients with acute febrile illness from the Peruvian coast. BMC Res Notes. 2020 Feb 10;13(1). doi: 10.1186/s13104-020-4937-1 32041646 PMC7011230

[26] Riley RD, Ensor J, Snell KIE, Harrell FE, Martin GP, Reitsma JB, et al. Calculating the sample size required for developing a clinical prediction model. BMJ [Internet]. 2020 Mar 18 [cited 2020 Nov 25];368. Available from: https://pubmed.ncbi.nlm.nih.gov/32188600/. doi: 10.1136/bmj.m441 32188600

[27] Valle-Mendoza J del, Vasquez-Achaya F, Aguilar-Luis MA, Martins-Luna J, Bazán-Mayra J, Zavaleta-Gavidia V, et al. Unidentified dengue serotypes in DENV positive samples and detection of other pathogens responsible for an acute febrile illness outbreak 2016 in Cajamarca, Peru. BMC Res Notes 2020 131 [Internet]. 2020 Oct 6 [cited 2021 Jul 6];13(1):1–7. Available from: https://bmcresnotes.biomedcentral.com/articles/10.1186/s13104-020-05318-5. doi: 10.1186/s13104-020-05318-5 33023645 PMC7541171

[28] Rojas A, Stittleburg V, Cardozo F, Bopp N, Cantero C, López S, et al. Real-time RT-PCR for the detection and quantitation of Oropouche virus. Diagn Microbiol Infect Dis [Internet]. 2020 Jan 1 [cited 2020 Jun 22];96(1). Available from: https://pubmed.ncbi.nlm.nih.gov/31727377/. doi: 10.1016/j.diagmicrobio.2019.114894 31727377 PMC6906250

[29] Naveca FG, do Nascimento VA, de Souza VC, Nunes BTD, Rodrigues DSG, da Costa Vasconcelos PF. Multiplexed reverse transcription real-time polymerase chain reaction for simultaneous detection of Mayaro, Oropouche, and oropouche-like viruses. Mem Inst Oswaldo Cruz. 2017 Jul 1;112(7):510–3. doi: 10.1590/0074-02760160062 28591313 PMC5452489

[30] Robinson ML, Manabe YC. Reducing Uncertainty for Acute Febrile Illness in Resource-Limited Settings: The Current Diagnostic Landscape. Am J Trop Med Hyg [Internet]. 2017 [cited 2021 Jul 6];96(6):1285. Available from: /pmc/articles/PMC5462561/. doi: 10.4269/ajtmh.16-0667 28719277 PMC5462561

[31] Ricapa-Antay F, Diaz-Melon K, Silva-Caso W, Valle LJ del, Aguilar-Luis MA, Vasquez-Achaya F, et al. Molecular detection and clinical characteristics of Bartonella bacilliformis, Leptospira spp., and Rickettsia spp. in the Southeastern Peruvian Amazon basin. BMC Infect Dis [Internet]. 2018 Dec 4 [cited 2021 Jul 6];18(1). Available from: /pmc/articles/PMC6280516/. 10.1186/s12879-018-3541-7 PMC6280516 30514235

